# High efficiency holographic Bragg grating with optically prolonged memory

**DOI:** 10.1038/srep36148

**Published:** 2016-10-26

**Authors:** Iam Choon Khoo, Chun-Wei Chen, Tsung-Jui Ho

**Affiliations:** 1 Pennsylvania State University, Electrical Engineering Department, University Park, PA 16802, USA

## Abstract

In this paper, we show that photosensitive azo-dye doped Blue-phase liquid crystals (BPLC) formed by natural molecular self-assembly are capable of high diffraction efficiency holographic recording with memory that can be prolonged from few seconds to several minutes by uniform illumination with the reference beam. Operating in the Bragg regime, we have observed 50 times improvement in the grating diffraction efficiency and shorter recording time compared to previous investigations. The enabling mechanism is BPLC’s lattice distortion and index modulation caused by the action of light on the azo-dopant; upon photo-excitation, the azo-molecules undergo transformation from the oblong-shaped Trans-state to the bent-shaped Cis-state, imparting disorder and also cause the surrounding BPLC molecules to undergo coupled flow & reorientation leading to lattice distortion and index modulation. We also showed that the same mechanism at work here that facilitates lattice distortion can be used to frustrate free relaxation of the lattice distortion, thereby prolonging the lifetime of the written grating, provided the reference beam is kept on after recording. Due to the ease in BPLC fabrication and the availability of azo-dopants with photosensitivity throughout the entire visible spectrum, one can optimize the controlling material and optical parameters to obtain even better performance.

There have been intense interests and research efforts to develop materials that meet many stringent requirements for holographic recording and display purposes such as high efficiency, acceptable writing/erasure/refresh speed and memory; fabrication and material processing cost are also important factors when wide spread usage is intended^1^,^2^,^3^,^4^,^5^,^6^,^7^,^8^,^9^,^10^. The recording media cover a wide range of crystals, soft matters, nano-particles and opto-mechanical assembly; processing and fabrication procedures vary from simple chemical synthesis or crystal growth and molecular self-assembly, to high-precision optical manipulation of nano-particles with pulsed lasers. State-of-the-art holographic materials developed so far, however, are still saddled with limitations or shortcomings of one kind or the other. For instance, fast acting materials generally possess short memory and therefore not suited for display, updatability or other image processing purposes, whereas those with long memory require complex and tedious processing during recording^5^, or extremely high electrical voltages and long writing/refresh times^7^,^8^.

Nonlinear optical materials such as inorganic crystals and semiconductors, organic photorefractive polymers and liquid crystals are naturally suited for direct-holographic recordings that involve light-light interaction mediated by the nonlinear optical response of the material^3^,^4^,^5^,^6^,^7^,^8^,^9^,^10^,^11^,^12^,^13^,^14^,^15^,^16^. In particular, owing to their fabrication ease and flexibility, and low optical power requirement associated with their highly nonlinear optical responses, inexpensive liquid crystals and organic polymers have been extensively studied^6^,^7^,^8^,^9^,^10^,^11^,^12^,^13^,^14^,^15^,^16^. There are of course shortcomings. Photorefractive polymers, for example, require a very high electrical voltage/field in all stages of recording and display^7^,^8^ while the birefringent nature of nematic and other liquid crystals^9^,^10^,^11^,^12^,^13^,^14^,^15^,^16^,^17^,^18^,^19^ mandates the use of polarized lights and restricts the fields-crystal interaction geometry. Moreover, nonlocal forces from boundary surfaces and un-activated regions in the liquid crystal become stronger as the cell thickness (d) and/or grating spacing (Λ) decreases, or when the liquid crystals are confined in highly restricted geometry such as micro-ring resonator, nano-structured fishnet metamaterials or photonic crystal inverse opals^18^,^19^,^20^,^21^. As a result, most holographic grating recording using nematic liquid crystals with visible light (λ ~ 0.5 μm) are conducted in the Raman-Nath regime (Λ^2^ ≫ λd) involving large grating constant (~10’ s μm) in a thin sample (up to ~100 μm). This regime tends to yield undesirable multi-order diffractions and low diffraction efficiency, and poor image resolution capability^14^. Attempts to operate in the Bragg regime (Λ^2^ ≪ λd) using *the same mechanism of optical field induced director axis reorientation* are also plagued with inefficiency due to these strong anchoring forces; when the wave mixing angle increases, corresponding to decreasing grating constant, the observed diffraction efficiency drops dramatically to vanishing value.

Here we demonstrate the possibility of circumventing these nematics’ limitations and obtaining dramatic improvement on all major performance factors by using mm-thick photosensitive azo-dye doped Blue-Phase liquid crystals (A-BPLCs) and operating in the hitherto unexplored *Bragg regime* (Λ^2^ ≪ λd). Blue-phase liquid crystals^22^,^23^,^24^,^25^,^26^,^27^,^28^,^29^,^30^,^31^,^32^ are essentially 3-D photonic crystals formed by natural molecular self-assembly. In the BPII and BPI phase, the doubly twisted cholesteric spirals arrange themselves into the respective simple cubic and BCC lattices with sub-optical-wavelength lattice constant, c.f. Fig. 1a–c. Bulk BPLC’s therefore exhibit *isotropic* index of refraction^24^ and generally polarization-insensitive responses. Because of the tightly bound molecules in the doubly-twisted cholesteric spirals, nonlinear response associated with molecular reorientation by optical field is generally weak in comparison to nematic. However, by incorporating photosensitive dyes that reside mainly in the defect region, c.f. Fig. 1d, a *more efficient* mechanism for index modulation associated with the action of light on the azo-dopant is manifested. In a yet-to-be optimized experimental setup, and using the same writing power as in previous studies^22^,^23^ of Raman-Nath grating, we have observed *polarization-independent* grating diffraction efficiency as high as 50% instead of ~1% and writing time of a few seconds instead of minutes. Furthermore, we have discovered a simple optical means of prolonging the lifetime of the written grating using the reference beam that is already in place; by keeping the reference beam on when the other beam (input signal) is turned off, the lifetime of the written grating/hologram can be *prolonged* from its natural relaxation time of a few seconds to several minutes.

As discussed in more details in the following sections, the underlying mechanism is BPLC’s lattice distortion and index modulation caused by the action of light on the azo-dopant. Upon photo-excitation, the azo-molecules transform from the oblong-shaped Trans-state to the bent-shaped Cis-state, causing disorder and the surrounding BPLC molecules to undergo coupled flow-reorientation process. These processes distort the A-BPLC crystalline lattice and produce an index of refraction change. We also show that the same mechanism of photo-induced Trans-Cis isomerism can also act to frustrate the free relaxation of the distorted lattice [written index grating] if the reference beam is kept on after recording, thus prolonging its lifetime.

The coherent holographic wave-mixing experimental set-up is shown in Fig. 2 [See also Method section]. Essentially two electronic-shutter modulated coherent 532-nm CW lasers beams are overlapped on the A-BPLC sample. The index grating produced by these writing beams is probed by a backward-propagating CW 633-nm laser in the phase-matched direction. The wave-mixing results obtained with the two dopants used are similar in many respects; here we focus on results and new insights into the underlying mechanism obtained with the more-stable CPND-57 azo-dopants.

## Results and Discussions

In general, strong single-order diffraction begins to manifest with a writing beam power on the order of a few mW; for the laser beam diameter of 3 mm, this power level corresponds to an intensity of several 10’s of mW/cm^2^. Typical dynamics of the diffraction signals for simultaneous turning on and off of the two writing beams are shown in Fig. 3a,b for two different writing times. Under a short writing time (~5 sec. or less), the diffraction is observed to build up to a maximum value towards the end of the excitation pulse, before decaying monotonically to vanishing value in ~5 sec, c.f. Fig. 3a. If the writing time is 8 seconds or longer, the diffraction is observed to reach the same maximum value in about the same time, but then exhibits a small abrupt upturn (lasting ~1 sec.) just when the writing beams are turned off, before resuming similar slow monotonic decay, c.f. Fig. 3b. The small abrupt rise is attributed to the fast decay of a small indexing component of opposite sign to the dominant contribution to lattice distortion, and is attributed to the *local disorder* caused by the bent-shaped Cis-isomers, which typically relax to the oblong-shaped Trans-state in ~1 sec^16^. On the other hand, the dominant component comes from lattice distortion associated with *slower rearrangement of the defect structure (disclination lines) associated with migration/flow of molecules* away from the high intensity regions (grating maxima) to the low intensity regions (grating minima); such processes take longer (~5 sec) to develop. Without further optimization, the peak diffraction efficiency is observed to be nearly 50% for writing beam powers of ~6 mW. In terms of the laser intensity (power per unit area), these power values correspond to intensities of ~70 mW/cm^2^ for the laser beam diameter of 3 mm. Under the Bragg condition (Λ^2^ ≪ λd, where Λ ~ 3 μm; thickness d = 1 mm, λ = 0.532 μm), the probe diffraction is sharply peaked at the wave-vector matching angle θ ~ 5.5° with a half-width of ~0.5°, as shown in Fig. 3c. The diffraction is also shown to be largely independent of the probe polarization relative to the writing beams’, manifesting the optical isotropy of the BPLC and the underlying mechanism that mediates the coherent optical wave mixing process.

Repeating these and other similar studies with various writing beam powers and durations, we observe that characteristically, the holographic grating takes just as long to decay as it takes to build up to the maximum, i.e. the memory lasts only as long as it takes to record; in fact, when the writing duration [when both beams are on] is longer than 40 seconds, the index grating begins to deteriorate due to several factors including migration of the dyes from the illuminated region and/or heating of the sample beyond to the isotropic phase [see later sections]. In order to create long-lasting grating, we have shown in a previous studies of azo (Methyl-red)-doped BPLC that application of a small DC field (~5 V/μm) during the holographic recording process can result in an index grating that will last almost indefinitely (days if left un-perturbed)^14^,^30^; in that case, however, complete erasure of the grating in order to record a new one has to be done by heating the sample through the isotropic liquid phase and cooling it back down to the Blue-phases - a procedure that takes up to a few minutes. Although the required electrical field (a few V/μm) is much smaller than the alarmingly high voltage (9000 V applied across a 100-μm thick cell) needed for similar operations with photorefractive polymers^7^,^8^, the refresh time of a few minutes remains an undesirable drawback of such field DC field induced memory storage effects in thses material systems.

We have discovered an all-optical means of significantly prolonging the memory of a grating written with short exposure time, while keeping the refresh time short. This comes about when the reference beam is kept on while the object beam is blocked, in our attempt to see if such uniform illumination could speed its relaxation and thus erase the written grating similar to holographic recording process in photorefractive crystals and polymers^3^,^7^,^8^. Instead, we observe that the reference beam actually prolongs the lifetime of the written grating, as illustrated in Fig. 4a–d. In general, absent the object beam after the recording, the diffraction signal continues to build up to a maximum and subsequently monotonically decays in a *much longer time than the free-relaxation.* In the BPII phase, such optically prolonged memory with the reference beam kept on lasts over 50 seconds compared to the 5 sec free relaxation time when both writing beams are turned off [c.f. Fig. 3a,b]. In the BPI phase, c.f. Fig. 4c,d, we have observed that the decay proceeds at a much slower pace than in the BPII case. In particular, if the grating is allowed to build up to the maximum before the object beam is turned off, the diffraction signal can be held at a steadily unchanged level for a long period until the reference beam is switched off, c.f. Fig. 4d. By varying the initial writing beam power and duration, we have found that this period of steady diffraction signal can last as long as 2 to 3 minutes before experiencing a slow decay to eventually vanishing value.

A plausible qualitative explanation for the prolonged persistence is as follows. When both writing beams are turned on, an *intensity grating* is imparted on the sample and generates a population grating of bent-shaped Cis-isomers which create local disorder at the intensity grating maxima (bright regions). At the same time the excess Cis-isomers in these intensity grating maxima migrate towards the intensity minima (dark regions) while relaxing to the oblong-shaped Trans-state, and cause the surrounding BPLC molecules in the defect regions to undergo coupled flow/reorientation. These local and nonlocal processes result in reconfiguration of the defect structure -thus forming a lattice distortion grating and the accompanying index grating to diffract the probe beam. *When both writing beams are then turned off*, the Cis-isomers returned to the ground Trans-state in ~1 sec^16^; such fast decaying local disorder is responsible for the observed small abrupt change (upturn) in the diffraction signal. Absent such Cis-isomers, the perturbed BPLC molecules undergo reverse flow-reorientation process to recover the initial defect structure in a characteristic lattice relaxation time of ~5 sec as depicted in Figs 3a,b and 4a–d.

However, if *only one of the writing beams is switched off,* the other beam that is kept on will now uniformly illuminate the entire region, and produce Cis-isomers in (previously)dark regions [grating minima] as well as in (previously) bright regions [grating maxima], albeit at a lower rate due to the reduced light level from one beam. Consequently the lattice distortion associated with the molecular movement and disorder caused by Cis-isomers continues to build up in both the bright and dark regions. Because of the prevailing different degree of lattice distortion between the bright and dark regions, i.e. an index grating formed just before the 1^st^ beam switch-off, the above mentioned lattice distortion build up in both regions effectively enhances the index grating as manifested by the continued build-up of the diffraction signals after the 1^st^ beam switch-off c.f. Fig. 4a–d. As the uniform illumination by the 2^nd^ beam continues, the index grating will reach a saturated level, i.e. maximum diffraction signal, when the lattice distortion in the bright region has fully developed while the distortion in the dark region is still developing. Subsequently, as the distortion in the dark region begins to ‘catch up’ with those in the bright region, the resulting index grating will be diminished. As shown in Fig. 4a,b for the BPII phase, and Fig. 4c,d for the BPI phase, under continuous illumination by the 2^nd^ laser, the time it takes for the lattice distortion to equilibrate (corresponding to vanishing index grating and diffraction signal), is considerably longer than the free-relaxation time when both beams are switched off. In this respect, we may regard the uniform illumination as an optical means to frustrate the free-relaxation of the lattice distortion and thus prolong the grating memory. Owing to the different defect structures in the BPI and BPII phase, the former being characterized by multiple separate disclinations which are prone to assume metastable configuration whereas the latter possesses continuous defect structure and undergoes direct transition from one configuration to another^31^,^32^, the time it takes for the system to approach final equilibrium configuration in the BPI phase is longer than in the BPII phase, c.f. Fig. 4a–d.

This scenario of molecules’ migration and flow/reorientation caused by laser excited Trans-Cis isomerism of the azo-dopant in A-BPLC’s under continuous illumination by a single pump beam is further supported by the following experiments on the transmission spectra, which depict how the photonic bandgap is modified by the laser induced lattice distortion. As shown in Fig. 5 for the case of 10-seconds illumination, the transmission dip is observed to shift from 530 nm to 560 nm; a much longer (1 minute) illumination achieves the same 30 nm shift but an overall increase in the transmission level. The spectral shift is due to lattice distortion and it reaches saturation in <10 sec, whereas the overall increase is attributed to azo-molecule migration away from high to low intensity region following the spatially varying intensity profile of the Gaussian laser beam. The difference in azo dye concentration also accounts for a different reflection color of the illuminated area (circular pattern) from the surrounding unexposed area, c.f. photo-inserts in Fig. 5 taken immediately after the pump beam is removed. The different-colored pattern is observed to persist for tens of seconds to minutes for sample maintained in the blue-phases, depending on the illumination duration and beam power. We note here that such massive dopant migration and changes in the sample reflection/transmission spectrum is not evident if the illumination time is short (<10 seconds) and the laser power is low (~a few mW or less). For holographic recording purposes, therefore, prolonged writing time and/or high beam power not only are unnecessary but also could lead to deleterious effects. For this particular sample, mW-power laser [beam diameter of 3 mm] with writing time of ~2–5 sec would suffice, and the best diffraction efficiency is obtained from sample maintained in the temperature regions around the BPI-BPII transition c.f. Fig. 6; operating too close to or above the isotropic temperature, or in the focal conic cholesteric phase yield vanishingly small diffraction signal. It is important to note here that the higher diffraction efficiency obtained in the BPII phase versus the BPI phase is due mostly to the temperature dependence of the photonic band-gap [transmission dip] of BPLC. In BPII phase (which reflects mostly in the blue region, c.f. Method section), the transmission of the green 532-nm laser is higher than the BPI phase [~50% versus ~10%]. Accordingly, for a given incident laser power, the effective writing power within the BPLC sample is higher in the BPII phase and thus yields a larger diffraction efficiency.

We have performed several holographic image processing feasibility demonstrations including optical wave-conjugation using the high diffraction efficiency and prolonged memory effect afforded by A-BPLC. For optical phase conjugation demonstration, an air force resolution chart is placed in the path of one of the writing beams to act as the object beam and a corrugated transparent plastic sheet is inserted in the path of the object beam before the sample to impart phase aberration. The reference beam, after passing the sample, is retro-reflected to probe (read) the hologram created by the reference and object beams, and generate the phase conjugated signal which counter-propagates along the object beam and passes through the aberration before being recorded on a screen. Figure 7a,b shows the input object beam without aberration and its reconstructed image (probe diffraction) which, due to the rather thick sample, shows some degree of degradation. The well-known ability^3^,^33^,^34^ of such optical phase conjugation process to produce a retro-reflected aberration-free image of the object beamis clearly illustrated by Fig. 7c,d for the input aberrated object beam and its reconstructed image, respectively. Despite the severe aberration suffered by the input object beam, the phase conjugated signal after reversing the course of the object beam through the sample and aberrator shows that the aberration has largely been compensated. Perhaps it is superfluous but important to point out that the reconstructed images (probe diffraction) also exhibit prolonged memory if the reference beam is kept on after the object beam is turned off; instead of switching off almost instantly in a few seconds when the object beam is blocked, the reconstructed image can be maintained for several minutes for easy recording or viewing purposes.

## Conclusion

We have demonstrated the possibility of recording Bragg grating with high diffraction efficiency using a Blue-phase liquid crystal constaining photosensitive azo-molecules. The principal underlying mechanism is lattice distortion initiated by photo-isomerization of the azo-dopant molecules that causes local as well as nonlocal disorder and lattice distortion. These operations require low writing beam powers on the order of a few mW (intensity on the orders of a few 10’ s mW/cm^2^) and writing/refresh times of a few seconds. We have also discovered a direct optical means of prolonging the lifetime of the written grating/hologram from a few seconds to minutes, by simply keeping the reference beam on after recording. The effect is explained by considering the dynamical interplay of various processes such as disorder, flow-reorientation and the specific defect structures of the BPLC lattice subjected to exposure to sinusoidal and/or uniform optical intensity. With optimization on several material and optical parameters such as dopant choice and concentration, operation wavelength regime, cell thickness, better quality mirrors and writing beam profile and coherence length, we expect to realize even better performance on various aspects of such first time demonstration of holographic recording with optically-controlled memory and image processing operation such as optical wave-front conjugation. Azo-dye doped BPLC’s thus present themselves as highly promising all-optical alternative to the current state-of-the-art materials for updatable holographic recording, display and image processing applications.

## Method

The starting mixture material for Blue-phase liquid crystal consists of a chiral dopant S-811 (Merck) dissolved in two nematic hosts with positive dielectric anisotropy, E48 and 5CB, in the ratio: E48 (32%): 5CB (32%): S811 (36%)^22^. An important advantage of Blue-phase liquid crystals (BPLC) over other ordered phases of liquid crystals such as nematic and cholesteric is that there is no need for surface alignment treatment when assembling the cell. Large area samples can be simply made by inserting isotropic liquid phase BPLC mixture between clean uncoated glass slides with plastic spacers (for thin samples), or filling flat glass cuvettes for the mm-thick BPLC cells used in our studies. As a function of temperature, pristine BPLC’s exhibit the following phases: transparent isotropic liquid phase for temperature above 31 °C; ***bluish color*** BPII phase (at ~31 °C–28 °C); ***greenish-color*** BPI (at ~28 °C–23 °C) and cholesteric focal conic phase N* (below 23 °C)^24^. In our studies, we have used two azo-dyes to enhance the nonlinear optical responses of BPLC: non-mesogenic Methyl-red dye [Sigma-Aldrich] used previously^22^ and a mesogenic compound^16^ CPND-57 [BEAM CO.]. The latter is a eutectic mixture of CPND-5 and CPND-7 azo dyes, 1-(2-Chloro-4-N-nalkylpiperazinylphenyl)-2-(4-nitrophenyl) diazenes, in the ratio 1:3. CPND-57 is known to be very stable^16^ under optical excitation compared to Methyl-red. These azo-dyes generally absorb in the blue-green spectral region. Dye-dopant concentration used in our study is guided by the desired sample transmission level at the writing beam wavelength (532 nm). For the 1-mm thick sample in the BPII phase, a small concentration of dyes (<0.1%) results in an absorption loss of ~50% at 532-nm, but negligible small absorption loss at the probe wavelength (633 nm). Samples with this level of transmission is found to yield very large diffraction efficiency.

**Figure 2:**
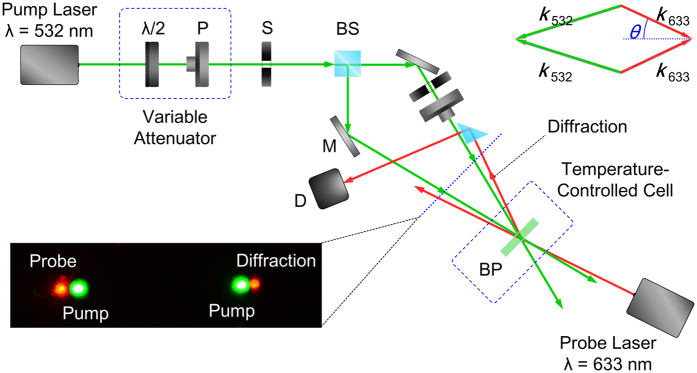
Experimental setup. Schematic depiction of the experimental setup for volume hologram grating formation and the wave vector (phase) matching condition for the four wave mixing process (shown in upper right corner) involving the two writing beams [wavelength = 532 nm] and the probe and its diffraction [wavelength of 633 (632.8) nm]. Here P: polarizer; BP: a-BPLC sample; S: mechanical shutter; BS: beam splitter; λ/2: half-wave plate; D: photodetector; M: mirror]. The photo insert on lower left corner shows the writing beams, the probe beam and the diffracted beam on the screen.

**Figure 3:**
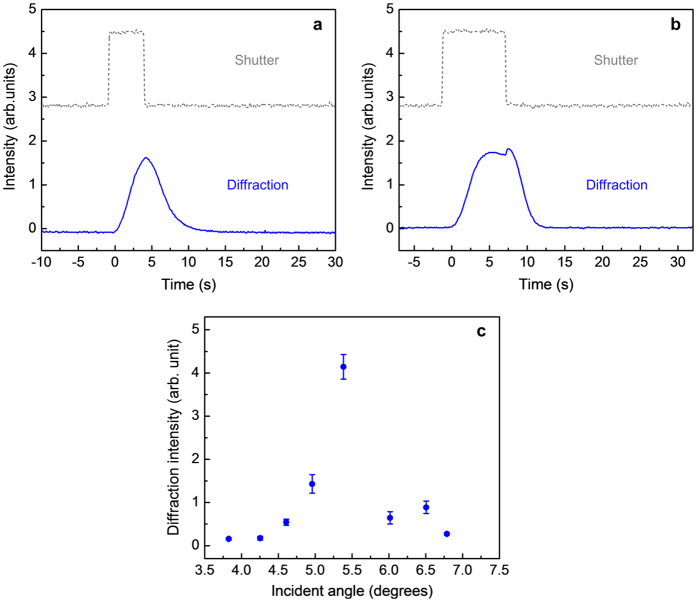
Switching dynamics with different writing duration and angular dependence of the probe diffraction. Typical dynamical evolution of the probe diffraction with step-on/off writing beams for beam powers: 3.2 mW and 3.7 mW; samples maintained in BPII phase. Similar results are obtained in BPI phase. (**a**) 5 s writing time, showing a smooth build up and decay (**b**) 8 s writing time, showing an abrupt increase when the writing beams are turned off. (**c**) Plot of the diffraction signal showing the sharp spike centered at the phase-matched angle.

**Figure 4:**
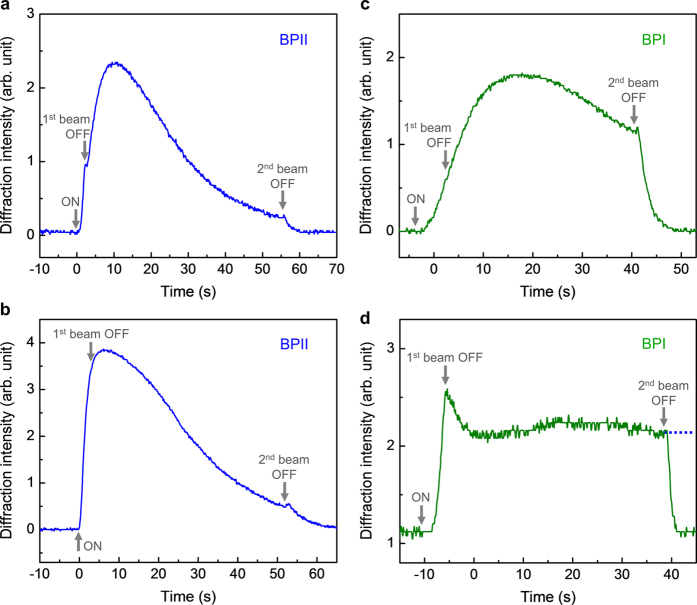
Dynamical dependence of the probe diffractions when the reference and object beams are switched off at different time. (**a**) BPII phase, one beam on for 2 seconds and switched off while the other beam is kept on for 55 seconds. Writing beam powers: 2.4 and 2.1 mW. (**b**) Same as (**a**) but for writing beam powers: 3.6 and 3.1 mW. (**c**) BPI phase, one beam on for ~6 seconds and switched off while the other beam is kept on for ~45 seconds. Writing beam powers: 2.4 and 2.1 mW. (**d**) Same as (**c**) but for writing beam powers: 3.6 and 3.1 mW. Dotted line indicates how the diffracted signal would persist for another 2 to 3 minutes if the reference beams is not switched off. In all cases, when the second beam is turned off, the diffraction signal disappears in <5 sec.

**Figure 5:**
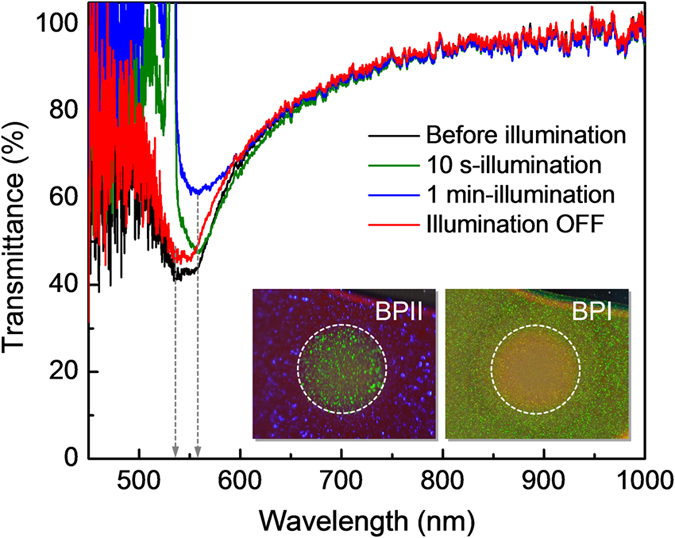
Transmission spectra of A-BPLC illuminated by a single pump laser. Transmission spectra exhibiting photonic bandgap (transmission dips) of the 1-mm thick A-BPLC sample illuminated by a 532-nm pump beam intensity ~15 mW/cm^2^ (pump power ~1.5 mW). The spectra are normalizing with the transmission spectrum of the sample in the isotropic (liquid) phase. Photo inserts show the different reflection colors from the regions under prolonged laser illumination at higher pump intensity ~80 mW/cm^2^ for BPI and BPII phase.

**Figure 6:**
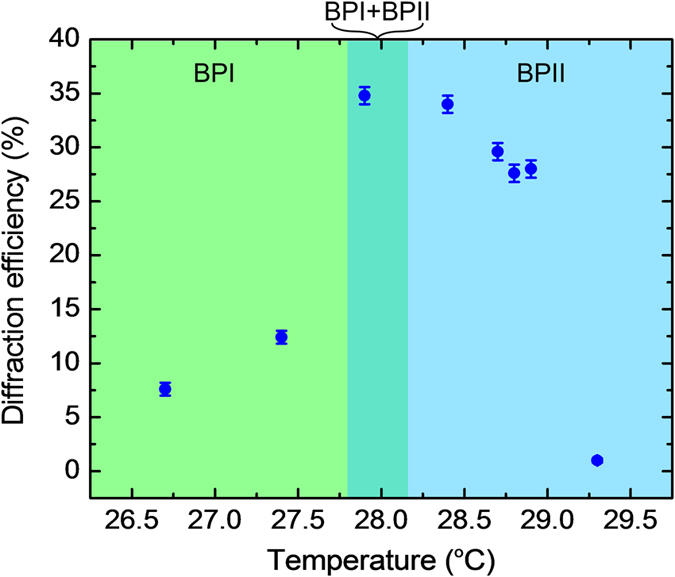
Temperature dependence of diffraction efficiency. Observed dependence of the maximum Bragg diffraction efficiency on the sample temperature in the BPI-BPII phases. Writing beam powers: 5.5 mW and 4.8 mW; writing time: 2 seconds.

**Figure 7:**
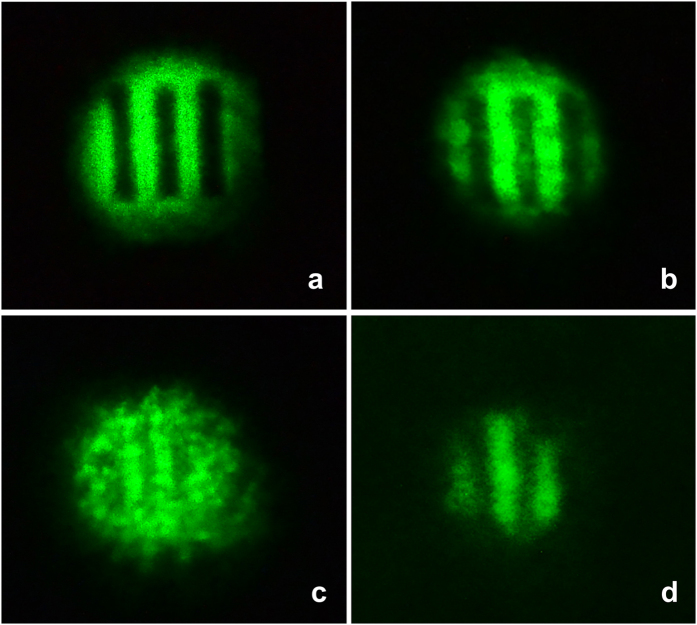
Phase conjugation demonstration. Optical wave-front conjugation otherwise known as real time holography demonstrating holographic image reconstruction with aberration correction capability using A-BPLC (**a**) Input object beam – dark bars; (**b**) Reconstructed image (**c**) Input object beam with severe aberration (**d**) Phase conjugated image showing the aberration has largely been corrected; dimmer signal is due to the scattering loss caused by the aberrator as well as transmission loss through the sample experienced by both the input object and the phase conjugated beams.

The optical wave-mixing experimental set-up consists of an electronically modulated/chopped CW (λ = 532 nm) laser beam that is expanded, collimated, and then divided into two coherent beams (the reference and object beams) of roughly equal power. These two writing beams are then overlapped on the sample at a wave mixing angle of 9.2 degrees, corresponding to a grating spacing Λ ~ 3 microns, i.e. operation in the Bragg grating (Λ^2^ ≪ λd). The Gaussian laser beam diameter is 3 mm. A CW He-Ne laser (λ = 633 nm and beam diameter of 2 mm) is used as a probe (reading) beam coming from the opposite side of the sample at the Bragg angle determined by the wave-vector matching diagram shown in the insert of Fig. 2. For optical wave front conjugation study, an air force resolution chart is placed in the path of one of the writing beams to act as the object beam and a corrugated transparent plastic sheet is inserted in the path of the object beam before the sample to impart phase aberration. The other writing beam acts as the reference beam and, after passing the sample, it is retro-reflected to probe (also termed read) the hologram created by the reference and object beams. The phase conjugated (sometimes termed real image) signal beam counter-propagates along the respective object beams.

## Additional Information

**How to cite this article**: Khoo, I. C. *et al*. High efficiency holographic Bragg grating with optically prolonged memory. *Sci. Rep.* **6**, 36148; doi: 10.1038/srep36148 (2016).

**Publisher’s note:** Springer Nature remains neutral with regard to jurisdictional claims in published maps and institutional affiliations.

## Figures and Tables

**Figure 1:**
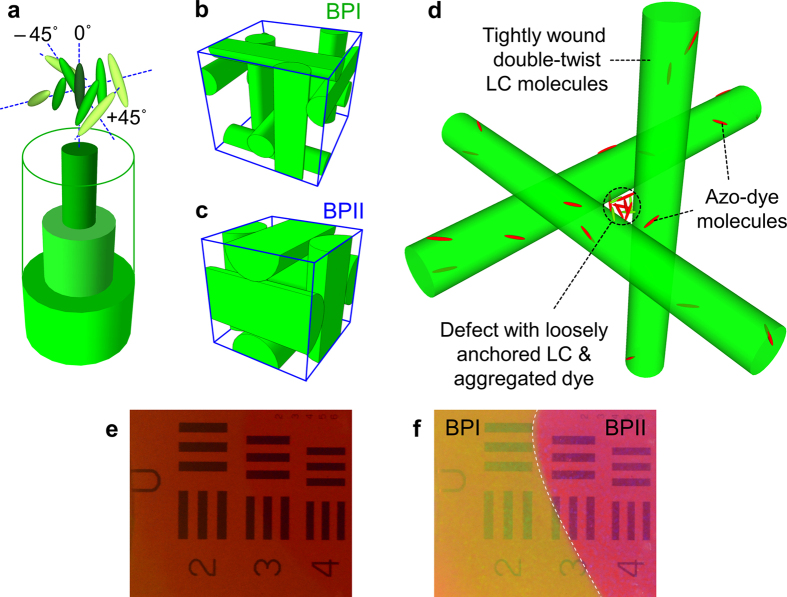
Molecular arrangement of liquid crystal blue-phases and selective reflections. Schematic depiction of the make-up of an azobenzene-doped blue-phase liquid crystal: (**a**) liquid crystal molecules in tightly wound double-twist cylinder; (**b**) BPI crystalline lattice; (**c**) BPII crystalline lattice; (**d**) enlarged view of the crystalline lattice and defect region where majority of dopant dye molecules aggregate, and participate actively in laser induced lattice distortion (**e**) Photograph of the BPLC placed on top of the Air force resolution chart; reddish coloration is due to the CPND-57 azo-dopant in BPLC; (**f**) Same as (**e**) but photo is taken with flash and exhibits more color display and contrast from the two blue-phase regions [regions of sample deliberately maintained at different temperatures].
